# Molecular characterization of carbendazim resistance of *Fusarium* species complex that causes sugarcane pokkah boeng disease

**DOI:** 10.1186/s12864-019-5479-6

**Published:** 2019-02-07

**Authors:** Shiqiang Xu, Jihua Wang, Haixuan Wang, Yixue Bao, Yisha Li, Muralidharan Govindaraju, Wei Yao, Baoshan Chen, Muqing Zhang

**Affiliations:** 1 State Key Lab for Conservation and Utilization of Subtropical Agric-Biological Resources, Guangxi University, Nanning, 530005 China; 2 Crop Research Institute of Guangdong Academy of Agricultural Science, Guangzhou, 510640 China

**Keywords:** Pokkah boeng, *Fusarium verticillioides*, *Fusarium proliferatum*, Carbendazim-induced mutant, Gene expression profile

## Abstract

**Background:**

Pokkah boeng is one of the most serious and devastating diseases of sugarcane and causes significant loss in cane yield and sugar content. Although carbendazim is widely used to prevent fungal diseases, the molecular basis of *Fusarium* species complex (FSC) resistance to carbendazim remains unknown.

**Results:**

The EC_50_ (fungicide concentration that inhibits 50% of mycelial growth) values of carbendazim for 35 FSC isolates collected in cane growing regions of China were ranged from 0.5097 to 0.6941 μg mL^− 1^ of active ingredient (a.i.), in an average of 0.5957 μg a.i. mL^− 1^. Among carbendazim-induced mutant strains, SJ51M (*F. verticillioides*) had a CTG rather than CAG codon (Q134L) at position 134 of the FVER_09254 gene, whereas in the mutant strain HC30M (*F. proliferatum*) codon ACA at position 351 of the FPRO_07779 gene was replaced by ATA (T351I). Gene expression profiling analysis was performed for SJ51M and its corresponding wild type strain SJ51, with and without carbendazim treatment. The gene expression patterns in SJ51 and SJ51M changed greatly as evidenced by the detection of 850 differentially expressed genes (DEGs). Functional categorization indicated that genes associated with oxidation-reduction process, ATP binding, integral component of membrane, transmembrane transport and response to stress showed the largest expression changes between SJ51M and SJ51. The expression levels of many genes involved in fungicide resistance, such as detoxification enzymes, drug efflux transporters and response to stress, were up-regulated in SJ51M compared to SJ51 with and without carbendazim treatment.

**Conclusion:**

FSC was sensitive to carbendazim and had the potential for rapid development of carbendazim resistance. The transcriptome data provided insight into the molecular pathways involved in FSC carbendazim resistance.

**Electronic supplementary material:**

The online version of this article (10.1186/s12864-019-5479-6) contains supplementary material, which is available to authorized users.

## Background

Pokkah boeng disease caused by FSC was firstly recognized more than 100 years ago and is a devastating disease that affects sugarcane production worldwide [1–3]. In recent years, pokkah boeng disease has become increasingly severe in China. Pokkah boeng causes serious yield losses (about 10~40%) in commercial sugarcane production. Disease outbreaks in susceptible cultivars have been reported in Yunnan, China and Shahjahanpur, India [2, 4]. The characteristic symptoms manifest as chlorosis, twisting and shortening of young leaves as well as stalk rot. Many *Fusarium* species, such as *F. moniliformae*, *F. sacchari*, *F. verticillioides* and *F. moniliforme* var. *subglutinans*, have been reported as causal organisms of pokkah boeng disease [2, 5–7]. *F. verticillioides* and *F. proliferatum* are two major *Fusarium* species that cause sugarcane pokkah boeng disease in China, with *F. verticillioides* accounting for over 90% of the recorded disease [8].

Methyl benzimidazole carbamate (MBC) fungicides, particularly carbendazim, a broad spectrum fungicide, provide effective control of fungal diseases in a variety of crops. Carbendazim can prevent pokkah boeng disease, especially in the top rot phase infection [9]. In China, carbendazim has been widely used to control pokkah boeng disease since 1980s, especially in chewing cane. However, carbendazim cannot be continuously applied to prevent pokkah boeng disease in chewing cane fields due to the development of resistant FSC. In our field survey, about 15% of carbendazim-resistant FSC was seen with carbendazim EC_50_ of 1.86 μg a.i. mL^− 1^ in a chewing cane field in Guangzhou, China, indicating that repeated and intensive applications of carbendazim could have promoted emergence of resistant FSC strains in this region. Thus, investigation of carbendazim sensitivity in FSC isolates from sugarcane planting areas in China is essential to assess the risk of carbendazim resistance.

MBC fungicides inhibit mitosis by binding to β-tubulin and inhibiting tubulin biosynthesis [10, 11]. MBC-resistant strains have been reported in many phytopathogenic fungi [12–15]. Point mutations in β-tubulin 2 (*Tub2*) can confer MBC resistance by altering amino acids in the MBC binding site [16]. Substitutions at codons 6, 50, 134, 165, 167, 198, 200, 235, 240, 241 and 257 in the *Tub2* gene have been shown to cause MBC resistance in field or laboratory isolates of several pathogenic fungi [16–19]. However, several exceptions have been reported wherein mutations that cause changes in *Tub2* amino acid sequences are not detected in several resistant strains, suggesting that there may be other pathways involved in the molecular mechanism of MBC resistance [20, 21].

Indeed, fungicide resistance can also be induced through various mechanisms, mainly structural alterations in target sites that reduce fungicide affinity, overexpression of fungicide target genes and metabolic decomposition and active efflux to reduce intracellular fungicide concentration [16, 22]. The development of genomic, proteomic, transcriptomic and bioinformatics provides a new strategy for exploring fungicide resistance mechanisms. RNA sequencing (RNA-seq) is an effective method to identify responses at the gene expression level and has been widely used in the research of resistance mechanisms. Indeed, transcriptome data reveal that *Trichophyton rubrum* response to acriflavine-involved genes categorize as oxidation-reduction reaction, transmembrane transport, and metal ion binding [23]. Genes encoding proteins involved in drug efflux confer *Penicillium digitatum* prochloraz resistance. These genes include transporters of the major facilitator superfamily (MFS), ATP-binding cassette transporters and the multidrug and toxic compound extrusion family [24].

The molecular basis of FSC resistance to carbendazim in different sugarcane planting areas around China is unclear, and carbendazim-resistant mutants have not been characterized. Therefore, the objectives of this study were to: (i) determine the carbendazim sensitivity of FSC isolates from different sugarcane planting areas around China, (ii) carry out a preliminary assessment of the risk of FSC resistance to carbendazim and characterize the carbendazim-induced mutants and (iii) understand the molecular mechanisms that could promote the development of carbendazim resistance in FSC.

## Results

### Sensitivity of FSC to Carbendazim

**Fig. 1:**
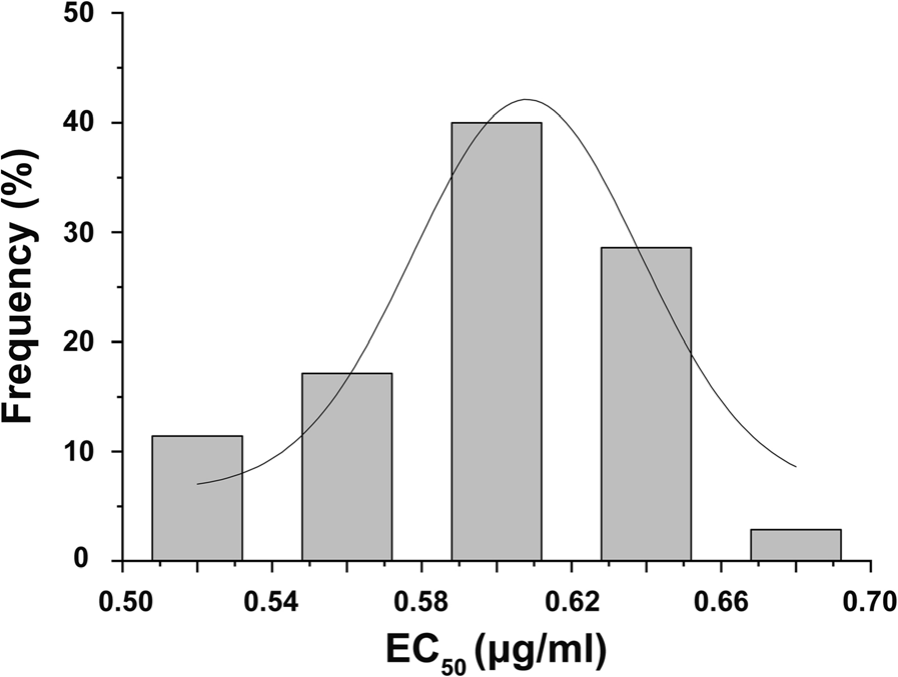
Frequency distribution of EC_50_ values to carbendazim for 35 FSC isolates. The isolates were recovered from major sugarcane production areas of China and the sensitivity curve was normally distributed over a sensitive range

None of the 35 FSC isolates grew on potato dextrose agar (PDA) amended with 1.2 μg a.i. mL^− 1^ carbendazim, but at 1.1 μg a.i. mL^− 1^ carbendazim, three isolates grew slowly (1 to 3 mm colony diameter). The carbendazim EC_50_ values for the 35 isolates ranged from 0.5097 to 0.6941 μg a.i. mL^− 1^ with an average EC_50_ of 0.5957 μg a.i. mL^− 1^ (Additional file 1: Table S1), indicating that these FSC isolates were susceptible to carbendazim. The normal distributions of EC_50_ of these isolates indicated that the 0.5957 μg a.i. mL^− 1^ was a suitable threshold concentration to assess carbendazim resistance in the subsequent experiments (Fig. 1).

### Carbendazim-induced mutants in vitro

**Fig. 2:**
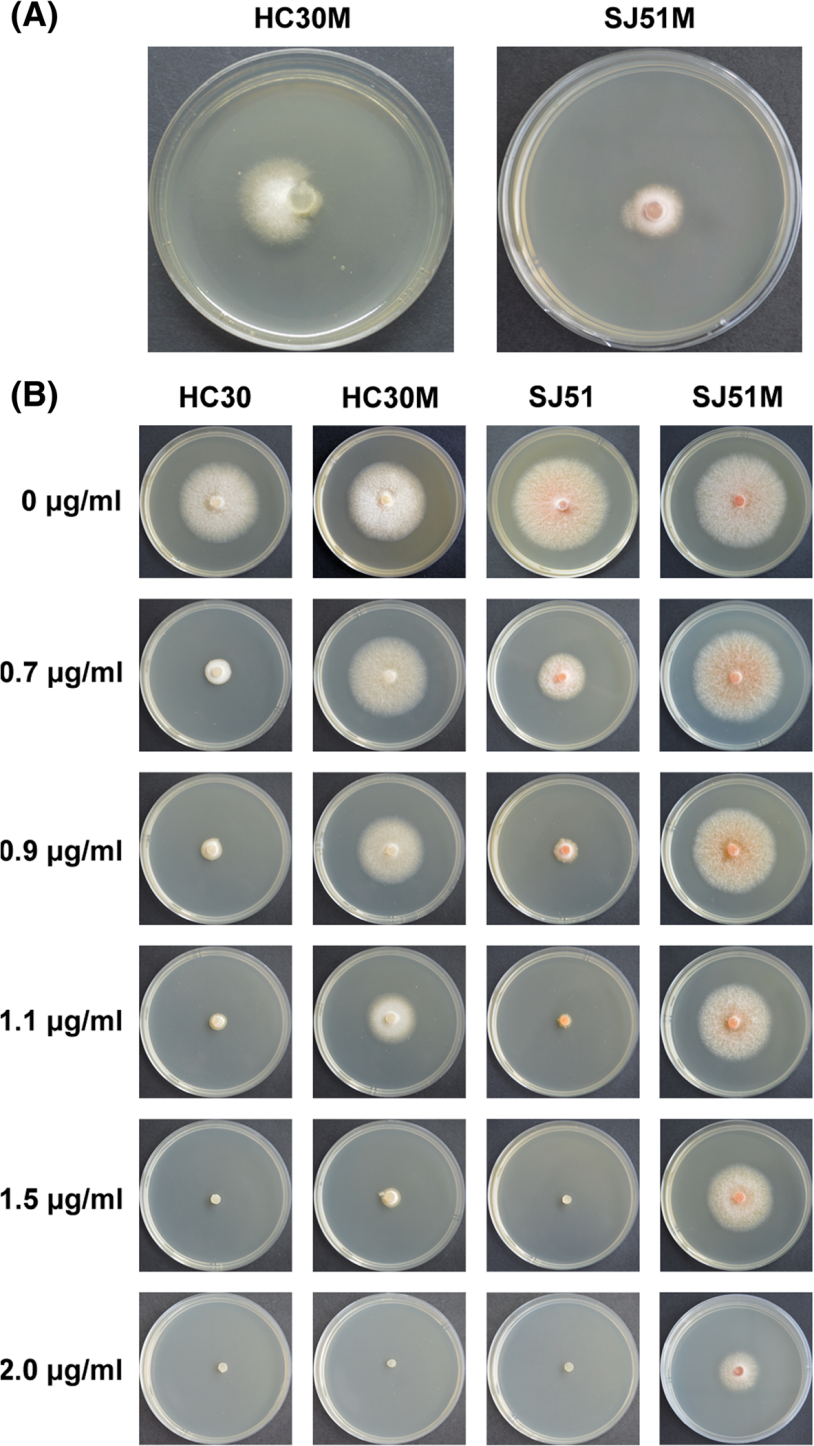
Colony morphology of FSC wild types and their mutants. (**a**) The fan-shaped region on the edge of the colony induced by carbendazim of SJ51 (on day 5) and HC30 (on day 8); (**b**) Mycelial growth of two FSC strains and their resistant mutants exposed to carbendazim. All strains were grown at 28 °C for 3 days on PDA media amended with carbendazim at 0, 0.7, 0.9, 1.1, 1.5 or 2.0 μg a.i.mL^− 1^

Isolates exposed to different carbendazim concentrations were continuously cultured at 28 °C in the dark to induce the rapid growth of mutants, which grew in a fan shape at the edge of some colonies. The earliest appearance of a mutant area occurred at the edge of a SJ51 colony on day 5 of culture (Fig. 2a). After 14 days of culture, 18 fan-shaped mutant areas were obtained from 14 strains at carbendazim concentrations ranging from 0.6 to 0.9 μg a.i. mL^− 1^. Three mutants were obtained from strain DH19, two from HC35 and LW54, and only one from the other 11 tested isolates. Carbendazim sensitivity of these mutants was measured after sub-culturing for 10 continuous generations on carbendazim-free PDA medium. Five mutants had higher and more stable resistance to carbendazim with EC_50_ > 1.0 μg a.i. mL^− 1^, whereas another 13 mutants had EC_50_ values that were similar to those for the wild type (Additional file 2: Table S2). The fan-shaped region and colony morphology exposed to different concentration of carbendazim of SJ51M and HC30M were presented in Fig. 2.

### Detection of mutations in *Tub2* genes in carbendazim mutants

Genome sequencing of *F. verticillioides* CNO-1 showed two genes encoding *Tub2* (FVER_05465 and FVER_09254). The coding regions of FVER_05465 had 1341 nucleotides encoding 446 amino acids, which had 100% homology to *F. sacchari* FRC R-6865 *Tub2* (GenBank accession number KU171789.1). Meanwhile, the coding regions of FVER_09254 included 1347 nucleotides that encode 448 amino acids, which were 99% homologous to that of *F. fujikuroi* (GenBank accession number AHG97571.1). *Tub2* DNA sequences were amplified and sequenced from five carbendazim-resistant mutants (SJ51M, HC30M, FZ15M, YN54M and FN22M) and the corresponding wild type strains. In the FVER_09254 gene from SJ51M, the CAG codon at position 134 was replaced by CTG (Q134L; A/T transition), whereas in mutant HC30M the codon ACA at position 351 of FPRO_07779 was replaced by ATA (T351I; C/T transversion) (Additional file 3: Figure S1). No point mutations in FVER_09254 and FVER_05465 were detected in the FZ15M, YN54M and FZ22M mutants.

### Temperature response and pathogenicity of carbendazim resistant mutants

The *Tub2* mutants were tested for their ability to grow at various temperatures with and without carbendazim. After culturing for 5 days, SJ51M grew at all tested temperatures (15 °C, 28 °C, 34 °C and 37 °C) on PDA with 1.9 μg a.i. mL^− 1^ carbendazim (nearly equal to EC_50_ of SJ51M), but wild type SJ51 failed to grow. The mutant HC30M grew at 28 °C and 34 °C on PDA amended with 1.2 μg a.i. mL^− 1^ carbendazim (nearly equal to EC_50_ of HC30M), whereas wild type HC30 failed to grow. Neither the mutant HC30M nor its wild type counterpart HC30 grew at 15 °C on PDA medium with carbendazim but did grow on PDA without carbendazim, whereas HC30M showed little growth at 37 °C in the presence or absence of carbendazim, similar to the growth of HC30 in absence of carbendazim (Additional file 4: Table S3). After culturing for 5 days at 28 °C on PDA medium, the radial growth (colony diameter) and colony morphology of SJ51M and HC30M were similar to wild type SJ51 and HC30 (Additional file 5: Figure S2). In pathogenicity assays, sugarcane plants inoculated with mutant (SJ51M and HC30M) or corresponding wild type strains (SJ51 and HC30) showed typical symptoms (e.g., growth point rot) 10 days after inoculation, while the control plants remained asymptomatic (Additional file 5: Figure S2). These results indicated that *Tub2* point mutations did not affect the growth and pathogenicity of the SJ51M and HC30M mutants.

### Gene expression profiles of carbendazim-resistant mutants exposed to carbendazim in vitro

**Fig. 3:**
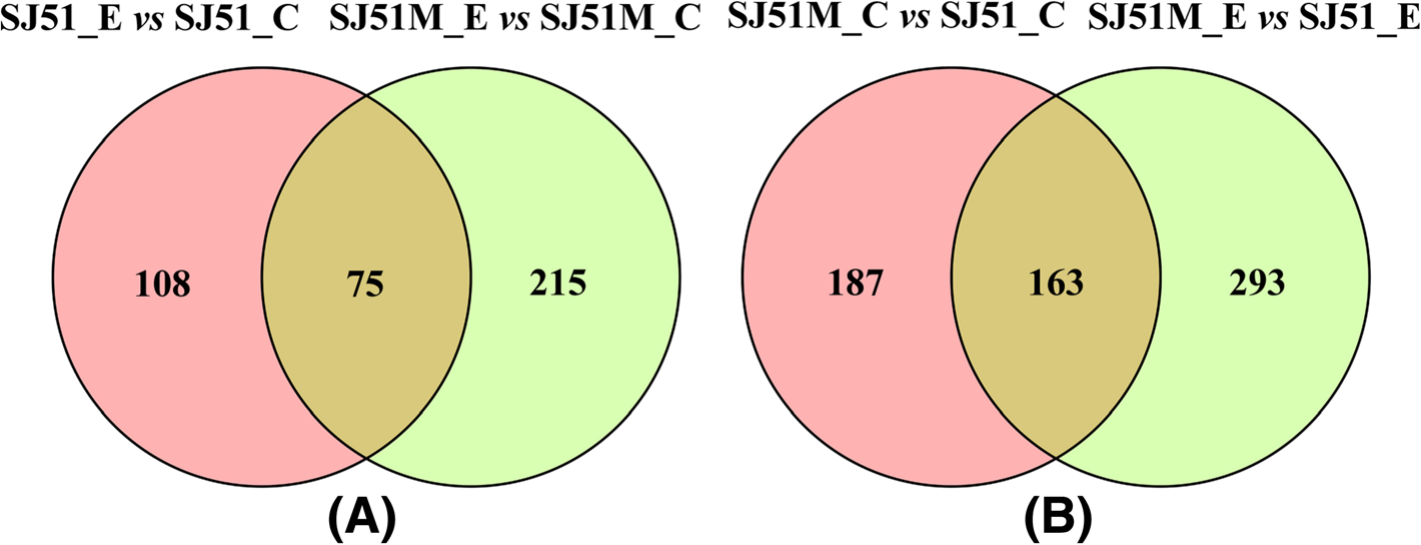
Venn diagrams showing the number of DEGs in SJ51M and its corresponding wild type strain SJ51. (**a**) The number of unique and shared DEGs of SJ51M and SJ51 responding to carbendazim treatment; (**b**) The number of unique and shared DEGs between SJ51M and SJ51 with and without carbendazim treatment. SJ51_C and SJ51M_C represented without carbendazim treatment, while SJ51_E and SJ51M_E represented exposed to carbendazim treatment. The cut-off limit of DEGs was less than 0.05 FDR and greater than 2-fold change

Gene expression changes in the carbendazim-resistant mutant SJ51M and wild-type SJ51 exposed to carbendazim were explored by Illumina sequencing. More than 229 million high-quality reads were generated from the samples and over 75% of the total reads mapped to the *F. verticillioides* CNO-1 genome (Additional file 6: Table S4). These data were deposited in the Sequence Read Archive (SRA) in the GenBank database under accession number SRP127969. Both SJ51 and the carbendazim-resistant mutant SJ51M grew on potato dextrose broth (PDB) medium amended with the corresponding carbendazim EC_50_ (1.87 a.i. mL^− 1^ and 0.61 a.i. mL^− 1^ for SJ51M and SJ51, respectively). A total of 290 DEGs were detected in SJ51M, including 225 and 65 that were up- and down-regulated, respectively. Wild type SJ51 showed 183 DEGs, including 135 up-regulated and 48 down-regulated genes (Table 1). SJ51 and SJ51M shared 75 DEGs after carbendazim exposure, whereas 215 unique DEGs were detected in mutant SJ51M and wild type SJ51 had 108 unique DEGs (Fig. 3a and b). Overall, 456 and 350 DEGs were detected between SJ51 and SJ51M with and without carbendazim treatment, respectively (Table 1 and Fig. 3). These results demonstrated that the gene expression patterns for SJ51 and SJ51M changed significantly under both normal conditions and carbendazim treatment, suggesting that some mechanisms may be specific to the development of carbendazim resistance.

**Table 1:** Number of DEGs in SJ51M and its corresponding wild type strain SJ51

DEG Set	All DEGs	Up-regulated DEGs	Down-regulated DEGs
SJ51_E vs SJ51_C	183	135	48
SJ51M_E vs SJ51M_C	290	225	65
SJ51M_C vs SJ51_C	350	183	167
SJ51M_E vs SJ51_E	456	278	178

SJ51_C and SJ51M_C represented without carbendazim treatment, while SJ51_E and SJ51M_E represented exposed to carbendazim treatment. The cut-off limit of DEGs was less than 0.05 FDR and greater than 2-fold change

**Fig. 4:**
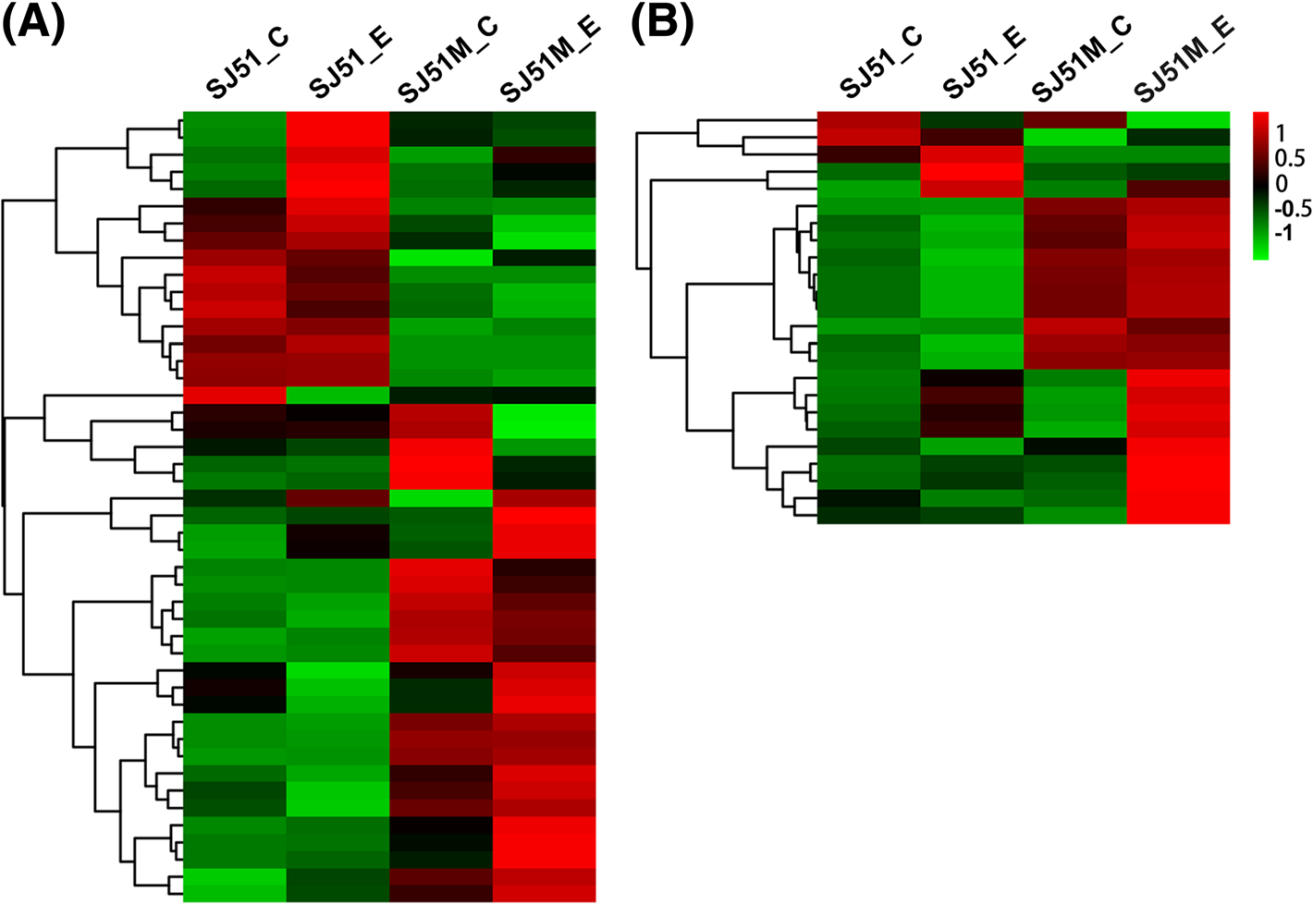
Heatmaps showing the expression pattern of DEGs. The expression pattern of DEGs related to transmembrane transport (GO:0055085) (**a**) and response to stress (GO:0006950) (**b**). SJ51_C and SJ51M_C represented without carbendazim treatment, while SJ51_E and SJ51M_E represented exposed to carbendazim treatment. Color scale showing the level of gene expression of log_2_ (FPKM+ 1). The cut-off limit of DEGs was less than 0.05 FDR and greater than 2-fold change

**Fig. 5:**
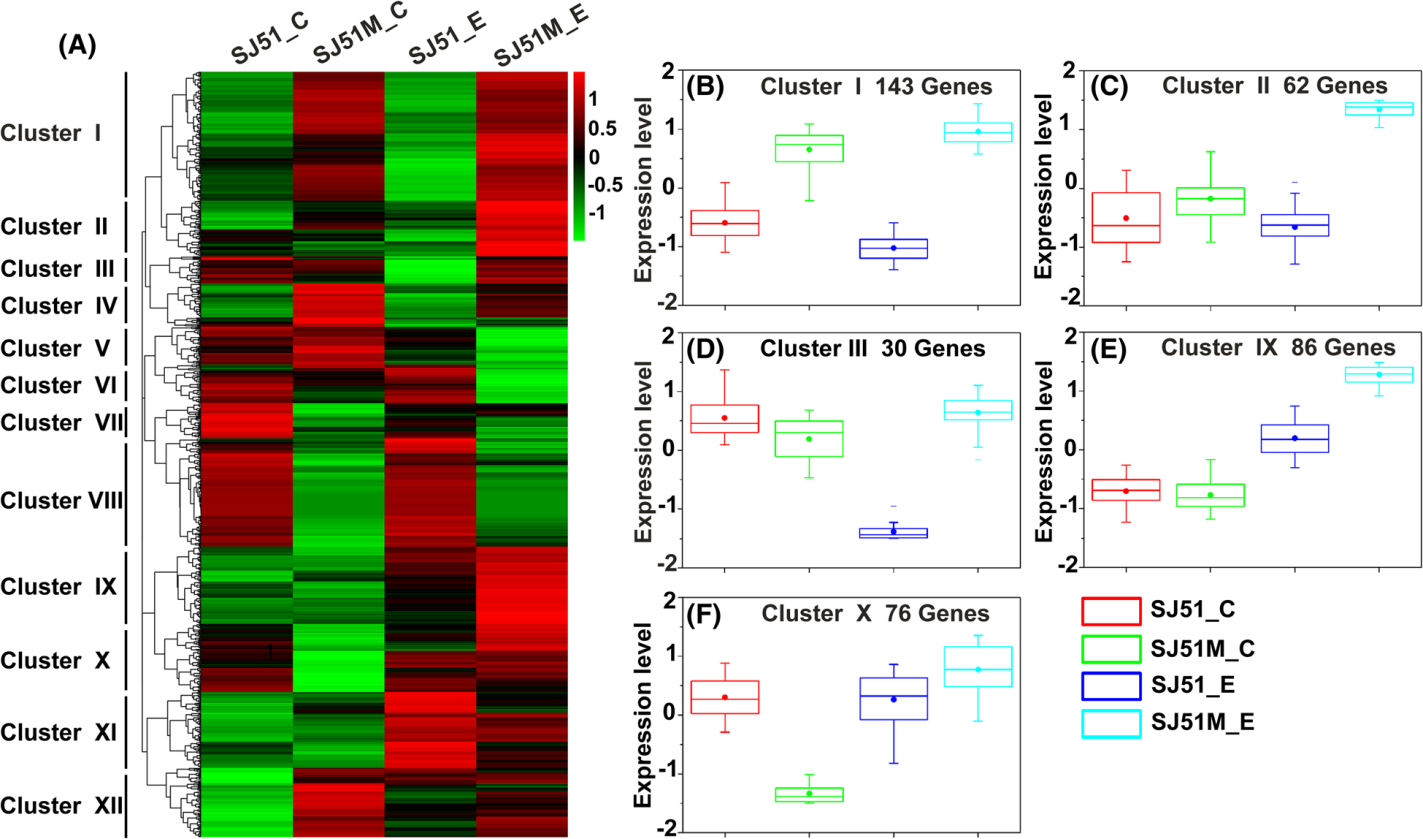
Hierarchical clustering showing the expression of carbendazim-induced DEGs. Gene expression pattern for 850 DEGs clustering into twelve clusters (**a**) and distribution of gene expression values with similar modulation in clusters I (**b**), II (**c**), III (**d**), IX (**e**) and X (**f**) which showed the increased expression in SJ51M_E compared with SJ51 after exposure to carbendazim. SJ51_C and SJ51M_C represented without carbendazim treatment, while SJ51_E and SJ51M_E represented exposed to carbendazim treatment. Color scale showed the level of gene expression of log_2_ (FPKM+ 1). The cut-off limit of DEGs was less than 0.05 FDR and greater than 2-fold change

To characterize how mutations in SJ51M affect carbendazim resistance, gene ontology (GO) enrichment analysis of the DEGs was performed using GOseq R packages (Additional file 7: Table S5). The function categories of oxidation-reduction process, ATP binding, integral component of membrane, transmembrane transport and response to stress were the most abundant in SJ51M relative to wild type SJ51. These results indicated that both energy metabolism and membrane stability/permeability were significantly affected by carbendazim. Most DEGs related to transmembrane transport (Fig. 4a) and response to stress (Fig. 4b) showed higher transcript levels in SJ51M compared to SJ51 under both normal conditions and in the presence of carbendazim treatment. To elucidate the similarities and differences in the expression pattern of the DEGs in the four treatments, a hierarchical clustering analysis was performed (Fig. 5a). The expression patterns of 850 DEGs could be divided into 12 clusters, suggestive of differences between SJ51M and SJ51 in response to carbendazim pressure. We focused our attention on the five clusters that contained genes with increased expression in SJ51M_E that were likely related to carbendazim resistance (cluster numbers I, II, III, IX and X, respectively). In cluster I (Fig. 5b), 143 genes had increased expression in SJ51M relative to SJ51 after exposure to carbendazim, including three genes (FVER_03030, FVER_09237 and FVER_11289) encoding the ABC multidrug transporter, which is critical for transmembrane transport during drug efflux. Nine genes belong to MFS, one of which encodes caffeine resistance protein 5 and two encode the HC-toxin efflux carrier TOXA. Increased expression was seen for FVER_09899, encoding a glutathione S-transferase that is important for fungicide detoxification. In addition, a group of genes related to response to stress and oxidative stress were identified in this cluster, and included heat shock protein, catalase and thioredoxin. In cluster II, IX and X (Fig. 5c, e and f), several genes were highly expressed during exposure of SJ51M to carbendazim, notably genes related to transmembrane transport, including four genes encoding the MFS transporter and one encoding the ABC transporter in cluster II. Two genes (FVER_05972 and FVER_12450) were related to glyoxalase/bleomycin resistance protein/dioxygenase superfamily in cluster IX. After exposure to carbendazim, FVER_12450 expression increased 3.19-fold in SJ51, whereas expressions of FVER_12450 and FVER_05972 showed increases of 10.77- and 16.11-fold in SJ51M, respectively. Intriguingly, two genes (FVER_08360 and FVER_05071) in cluster X that were related to the kinesin family play a critical role in mitosis by mediating microtubule assembly. Thirty genes in cluster III (Fig. 5d) had strongly decreased expression in wild type SJ51 upon exposure to carbendazim, but showed slightly up-regulated expression in the resistant mutant SJ51M, indicating that several cellular processes kept normal at the mutants of SJ51M exposed to carbendazim. In this cluster, FVER_09965 encoding kinesin-related protein KIP3, and FVER_14552 encoding GTPase-binding protein rid1, were observed.

### Quantitative real-time PCR (qRT-PCR) validation of target genes

**Fig. 6:**
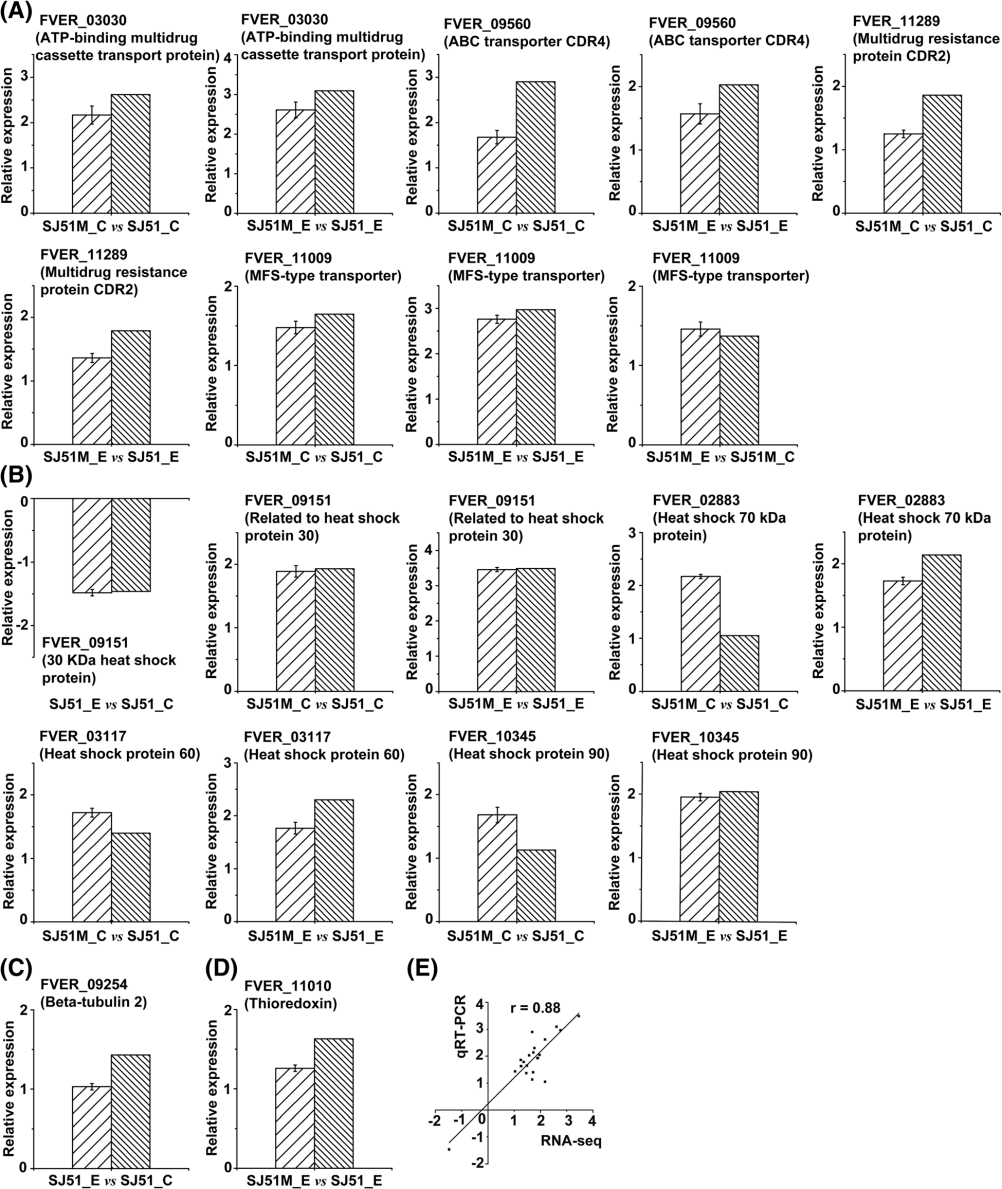
Validation of the DEGs expression exposed to carbendazim using qRT-PCR. Ten genes associated with transmembrane transport (**a**), response to stress (**b**), the target gene of carbendazim (**c**) and oxidoreductase activity (**d**), were selected for qRT-PCR analysis. (**e**) Pearson correlation of fold change analyzed between qRT-PCR and RNA-Seq. SJ51_C and SJ51M_C represented without carbendazim treatment, while SJ51_E and SJ51M_E represented exposed to carbendazim treatment. The mRNA abundance was normalized using the housekeeping actin gene, and the gene relative expression levels are represented by the log_2_Ratio. Data of qRT-PCR are presented as mean ± SD (*n* = 9)

The RNA-seq results were validated using qRT-PCR for ten genes selected for their involvement in transmembrane transport, oxidoreductase activity, response to stress and the target gene of carbendazim, including three ABC multidrug transporters (FVER_03030, FVER_09560 and FVER_11289), a MFS-type transporter (FVER_11009), four heat shock proteins (FVER_02883, FVER_03117, FVER_09151 and FVER_10345), a thioredoxin protein (FVER_11010) and the target gene *Tub2* (FVER_09254). Among these genes, expression of FVER_11009 (Fig. 6a) was up-regulated in SJ51M after carbendazim treatment, while expression of FVER_09151 (Fig. 6b) was down-regulated and FVER_09254 (Fig. 6c) was up-regulated in SJ51. FVER_11010 expression was up-regulated in SJ51M relative to SJ51 exposed to carbendazim (Fig. 6d). Three ABC multidrug transporters, the MFS-type transporter and four heat shock proteins were all up-regulated in SJ51M compared to SJ51 (Fig. 6a and b). The correlation between RNA-Seq and qRT-PCR was statistically significant (r = 0.88, *p* < 0.001) (Fig. 6e), suggesting that the transcriptome data were reliable and could provide a basis to explore the mechanism of FSC carbendazim resistance.

## Discussion

Carbendazim-resistant strains have been identified in chewing cane, which is widely used to prevent pokkah boeng disease [25]. Here we tested 35 FSC isolates recovered from major sugarcane production areas of China. To our knowledge, this is the first report to assess carbendazim sensitivity of FSC collected from different locations in China. All strains in this study were sensitive to carbendazim, with EC_50_ values ranging from 0.5097 to 0.6941 μg a.i. mL^− 1^ and a mean of 0.5957 μg a.i. mL^− 1^ (Additional file 1: Table S1). These results were similar to those for *Gibberella zeae* (a *F. graminearum* teleomorph), a carbendazim-sensitive strain having a mean EC_50_ value of 0.59 μg a.i mL^–1^ [26]. No resistant strains were detected in our collected isolates because carbendazim was more commonly used to treat seed cane prior to planting rather than for manual foliar application during the early stage of field production, such that mostly 3–7 month old sugarcane is infected in China. More strains will be collected and recovered from the field samples to test their sensitivity to carbendazim. With the higher incidence, few resistant cultivars were available, thus fungicides are needed to prevent pokkah boeng disease. Carbendazim was effective against pokkah boeng disease and had the lowest EC_50_ value relative to other fungicides (e.g., dimethachlon, chlorothalonil, mancozeb and Meroif™) [27]. Based on this analysis, it was presumed that carbendazim could be widely used to prevent pokkah boeng disease in China.

Carbendazim resistance develops rapidly in many pathogenic fungi [12–15]. Under selection pressure of fungicides, resistant strains can adapt to environmental conditions to become the major strain in pathogen populations, and in turn decrease fungicide effectiveness [16]. Although UV irradiation was widely used to select for fungicide resistance, in this study we instead used fungicide-induced mutations to assess the risk from emergence of resistant strains because mutants might better represent those that arise following fungicide application in the field. Here, fan-shaped growth of 18 mutants was induced at the edge of 14 isolates. After stability and sensitivity testing, the EC_50_ of the five mutant strains (SJ51M, HC30M, FN22M, YN54M and FZ15M) exceeded 1.0 μg a.i. mL^− 1^, which was higher than that of the corresponding wild type strain, and in the case of SJ51M, the difference in EC_50_ was 3-fold (Additional file 2: Table S2 and Fig. 2). A single application of carbendazim could quickly induce resistant strains in vitro, indicating that FSC can easily develop carbendazim resistance, even if the resistance level of the resistant isolates was not high. Compared to the other pathogenic fungi, strains of *F. graminearum* with a minimum inhibitory concentration of over 1.4 μg mL^− 1^ carbendazim were regarded as resistant isolates [14]. We observed similar trends in resistance level in mutant strains of FSC, which may be related to the characteristics of FSC.

The mechanism of resistance to carbendazim was associated with point mutations in the *Tub2* gene that change the structure of the fungicide binding site to decreases sensitivity in turn [16, 28]. According to the genome sequencing information, *F. verticillioides* CNO-1 has two homologous *Tub2* genes. A point mutation at codon 134 (Q134L) was detected in FVER_09254 from the resistant strain SJ51M; a similar mutation was reported in a laboratory-induced mutant of *Aspergillus nidulans* that had a mutation at codon 134 (Q134K) and was sensitive to heat, which could interfere with fitness under field conditions [17]. SJ51M with a mutation site at codon 134 was not heat sensitive and grew at 34 and 37 °C on PDA amended with carbendazim (Additional file 3: Figure S1 and Additional file 4: Table S3). A novel point mutation at codon 351 (T351I) was detected in FPRO_07779 in mutant HC30M (Additional file 3: Figure S1). A point mutation at codon 351 in the *Tub2* gene that confers carbendazim resistance has not been reported in other phytopathogenic fungi, either in field or laboratory isolates. The mutant HC30M carrying a mutation at codon 351 was cold sensitive and showed no resistance to carbendazim at 15 °C (Additional file 4: Table S3). We also confirmed the absence of mutations in the *Tub2* sequence in the resistant strains FN22M, YN54M and FZ15M. These results indicated that other mechanisms must be involved in FSC carbendazim resistance. The virulence of resistant isolates SJ51M and HC30M both induced disease to similar levels relative to the corresponding wild type strains SJ51 and HC30, respectively. Similar studies describing isolates of carboxin-resistant *Ustilago nuda* and boscalid-resistant *Alternaria alternata* also showed that the pathogenicity of these mutants was not significantly altered on *Hordeum vulgare* and *Pistachio*, respectively [29, 30].

Kinesins play important roles in transporting organelles and vesicles along microtubules and participate in cell mitosis [31]. The disorder of microtubule cytoskeleton and actin cytoskeleton affected mycelial growth and led to cell death in *F. graminearum* [32]. Interestingly, several genes involved in the kinesin family have been implicated in fungicide resistance. Here, expression of FVER_09965 (in cluster III) was sharply down-regulated in SJ51 but was slightly up-regulated in SJ51M after exposure to carbendazim. Expressions of FVER_05071, FVER_08360 and FVER_10733 were up-regulated in SJ51 and SJ51M after exposure to carbendazim, but the up-regulation was more pronounced in SJ51M (Table 2). These data indicated that *Tub2* mutations might affect microtubule structure that in turn affected kinesin function, especially after carbendazim treatment. Small GTPase family regulate a variety of signal transduction pathways, such as cytoskeletal formation and protein trafficking and endocytosis, which may play important roles in mediating cellular resistance to the platinum compound [33]. The Rho GTPases subfamily plays crucial roles in in regulating cytoskeletal organization and responding to extracellular growth factors [34]. The increased expression of FVER_00402 (encoding GTP-binding protein rhoC) upon exposure of SJ51M to carbendazim suggested an important role for signal transduction mediated by small GTPases during stress conditions (Table 2).

**Table 2:** The major genes modulated in SJ51M and its corresponding wild type strain SJ51 after carbendazim exposure

Gene ID	Log_2_ (Fold change)				Gene annotation
	SJ51_E vs SJ51_C	SJ51M_E vs SJ51M_C	SJ51M_C vs SJ51_C	SJ51M_E vs SJ51_E	
FVER_05972	–	4.0095^a^	–	2.2519^a^	Glyoxalase/Bleomycin resistance pro- tein/Dioxygenase superfamily
FVER_12450	1.6720^a^	3.4288^a^	0.1681	1.9312^a^	Glyoxalase/Bleomycin resistance pro- tein/Dioxygenase superfamily
FVER_11289	−0.1793	− 0.2528	1.8589^a^	1.7887^a^	Multidrug resistance protein CDR2
FVER_09237	−0.6985	0.6653	1.0074	2.2519^a^	Multidrug resistance protein fnx1
FVER_12798	−0.3570	−1.5140^a^	0.7393	−0.4143	Multidrug resistance protein CDR1
FVER_09560	0.0170	−0.8521	2.8984^a^	2.0326^a^	ABC transporter CDR4
FVER_04236	−0.0264	− 0.4497	1.0549^a^	0.6350	Probable MFS multidrug resistance transporter
FVER_10334	2.5884^a^	1.2424	1.3038	− 0.0260	Pleiotropic ABC efflux transporter of multiple drugs
FVER_03030	−0.3064	0.1645	2.6240^a^	3.0980^a^	Related to ATP-binding multidrug cassette transport protein
FVER_05891	0.0651	−0.3041	1.2302^a^	0.8643	Probable ATP-binding multidrug cassette transport protein
FVER_03551	0.1228	1.1575^a^	0.2902	1.3278^a^	ABC transporter ATP-binding protein
FVER_11889	−0.3146	0.0465	2.5807^a^	2.8235^a^	Caffeine resistance protein 5
FVER_01165	2.6628^a^	2.3188^a^	1.3811	1.0704 ^a^	Metal resistance protein YCF1
FVER_03117	−0.9540	−0.0568	1.4012^a^	2.3016 ^a^	Heat shock protein 60, mitochondrial
FVER_04661	−0.7160	0.4776	1.2237^a^	2.4205^a^	Heat shock protein 78, mitochondrial
FVER_04571	−0.6983	0.2399	1.1257^a^	2.0672^a^	Probable heat shock protein HSP104
FVER_12593	−0.3875	0.1239	0.9569	1.4716^a^	Heat shock protein sti1 homolog
FVER_02883	−0.7821	0.3016	1.0488^a^	2.1358^a^	Heat shock 70 kDa protein
FVER_09674	0.0077	0.1043	0.9416	1.0415^a^	Heat shock 70 kDa protein
FVER_01860	−1.1244^a^	0.5785	0.9860	2.6906^a^	Probable FES1-Hsp70 nucleotide exchange factor
FVER_11673	−0.9108	0.6567	0.7528	2.3235^a^	Heat shock 70 kDa protein
FVER_10345	−0.6977	0.2154	1.1314^a^	2.0537^a^	Heat shock protein 90
FVER_09151	−1.4554^a^	0.1075	1.9281^a^	3.4943^a^	Related to heat shock protein 30
FVER_04255	0.1770	−0.5574	1.1400^a^	0.4186	Probable chaperone protein HSP31
FVER_08232	−0.2435	0.5509	−1.0321^a^	− 0.2344	Catalase
FVER_08876	−0.1428	0.4837	1.5837^a^	2.2136^a^	Catalase
FVER_11010	−0.6185	0.1034	0.9058	1.6309^a^	Thioredoxin
FVER_02416	−0.1779	2.1616^a^	− 0.1494	2.1632^a^	Related to thioredoxin reductase
FVER_07248	−0.6687	−0.7614	1.1560^a^	1.0657^a^	Related to DSBA-like thioredoxin domain protein
FVER_13986	0.4550	1.4076^a^	0.3056	1.2617^a^	Related to cytosolic Cu/Zn superoxide dismutase
FVER_05859	1.2131^a^	0.5427	1.2630^a^	0.5976	Cytochrome P450 52A3-A
FVER_11192	−0.0087	0.0414	1.6595^a^	1.7130^a^	Cytochrome P450 52A6
FVER_08550	1.0365	−1.0307	2.2312^a^	0.1358	Related to Glutathione S-transferase II
FVER_09899	0.0960	−0.2003	1.5924^a^	1.2997^a^	Related to microsomal glutathione S-transferase 3
FVER_00097	−0.4491	−0.5106	1.4609^a^	1.4023^a^	Glutathione S-transferase
FVER_09254	1.4333^a^	0.3586	0.2223	− 0.8490	Beta-tubulin 2
FVER_09965	−1.6756^a^	0.3958	−1.3325	0.7231	Probable kinesin-related protein KIP3
FVER_10733	3.9528^a^	2.0084^a^	0.1825	−1.7512^a^	Kinesin-like protein KIP3
FVER_05071	0.2857	1.1278^a^	− 0.7088	0.1354	Kinesin-like protein klpA
FVER_08360	0.6639	3.7064^a^	− 1.4850^a^	1.5549^a^	Kinesin family member 1/13/14
FVER_00402	−0.3106	1.6102^a^	− 0.6439	1.2774^a^	GTP-binding protein rhoC

^a^Indicateed significantly differential expression. SJ51_C and SJ51M_C represented without carbendazim treatment, while SJ51_E and SJ51M_E represented exposed to carbendazim treatment. The cut-off limit of DEGs was less than 0.05 FDR and greater than 2-fold change. -, no expression was detected in SJ51_C and the expression abundance was very low in SJ51_E

Overexpression of drug target genes is also one mechanism that can confer resistance. This resistance mechanism involves a dose-effect, in which increased expression of the target gene can avoid saturation in the presence of a combination of fungicides [35]. Up-regulation of *Tub2* gene expression is associated with carbendazim resistance in *Paecilomyces lilacinus*, wherein the expression of *Tub2* is 4-fold higher than that in the wild type strain Pl36–1 [28]. Here, FVER_09254 expression was up-regulated by 2.7-fold in SJ51, but expression in SJ51M was similar in the presence or absence of carbendazim (Table 2). The compensation effect in SJ51 and the amino acid substitution Q134L in SJ51M might reduce the effects of a combination of carbendazim exposure and tubulin dysfunction.

A reduction in the concentration of toxic substances in cells mediated by overexpression of genes encoding detoxification enzymes and efflux transporters is correlated with drug resistance in several fungi [24, 36]. Cytochrome P450 monooxygenases (P450s) are known to mediate detoxification of fungicides, herbicide, pesticide and xenobiotics [37]. P450-mediated detoxification processes share common mechanisms and can also result in resistance of insects to insecticides [38, 39]. In a hypersensitive strain of *Candida albicans*, *CaALK8* (belonging to the CYP52 gene family) confers drug resistance [40]. The expressions of two genes (FVER_05859 and FVER_11192) related to CYP52 were up-regulated in SJ51M relative to wild type SJ51 (Table 2). Glutathione S-transferases (GSTs) involved in many essential cellular processes (e.g., xenobiotic detoxification, attenuation of oxidative stress, and signal transduction) have been reported to be associated with several resistance mechanisms. [41, 42] Our data showed that in the presence of carbendazim, the expressions of three genes (FVER_00097, FVER_08550 and FVER_09899) encoding GST were up-regulated in resistant strain SJ51M relative to wild type SJ51. Remarkably, two major transmembrane transporters in fungal efflux systems (ABC transporters and MFS transporters) have been reported to modulate fungicide sensitivity and resistance [43, 44]. Substrates of these transporters include endogenous or exogenous toxic components, such as fungicides and secondary metabolites from the cell. Several genes encoding ABC multidrug transporters and MFS multidrug transporters were identified from the transcriptome data (Table 2). Most were up-regulated in mutant SJ51M compared to wild type SJ51 in the presence of carbendazim treatment, especially the ABC multidrug transporters. A similar study was reported in the prochloraz-resistant strain HS-F6 [24]. Interestingly, genes related to drug detoxification and efflux transporters remained highly expressed after exposure to carbendazim even after continuous sub-culturing for 10 generations on fungicide-free PDA medium (in group SJ51M_C vs SJ51_C). These gene expression patterns might also occur under field conditions and could cause the rapid emergence of carbendazim resistant strains. The function of glyoxalase/bleomycin resistance protein/dioxygenase superfamily is to relieve the toxicity of methylglyoxal, a by-product of glycolysis [45]. The expression of two genes (FVER_05972, FVER_12450) associated with glyoxalase/bleomycin resistance protein/dioxygenase superfamily was intensely up-regulated in SJ51 and SJ51M after carbendazim treatment, especially in SJ51M (Table 2). It was speculated that this result might be related to dysfunctional energy metabolism in the presence of carbendazim and that the mutant SJ51M could have higher detoxification activity.

Expression of stress adaptation genes induced by exposure to drugs can overcome toxic effects and maintain cellular homeostasis that promotes survival. Many studies have demonstrated that cell stress responses and other mechanisms are associated with fungal resistance to azoles [46, 47]. The molecular chaperone heat shock 90 kDa protein (Hsp90) maintains protein stability to provide a critical mechanism for azole tolerance and resistance [48–50]. Here, expression of FVER_10345, which encodes an Hsp90, was up-regulated in SJ51M in the presence of carbendazim (Table 2). Notably, expressions of many genes encoding other kinds of heat shock proteins, such as Hsp70 (FVER_02883, FVER_09674, FVER_01860 and FVER_11673) and Hsp104 (FVER_04571), were also up-regulated in the SJ51M resistant mutant, indicating that many proteins were likely protected in the presence of carbendazim (Table 2). Similar results were observed for Hsp70 and Hsp104, which can induce protective responses to ketoconazole and amphotericin B in *Trichophyton rubrum* [51]. Expressions of two genes encoding thioredoxin and catalases were up-regulated in response to carbendazim in SJ51M. This up-regulation could protect the cell against oxidative stress and the accumulation of reactive oxygen species (ROS). These findings suggested that the mutant strain SJ51M could have higher viability following exposure to carbendazim. All these genes together enhanced survival of FSC exposed to carbendazim, and thus could be considered to be potential drug target genes.

## Conclusions

The results presented here showed that FSC were sensitive to carbendazim. Laboratory-induced resistant mutants obtained through carbendazim exposure indicated that FSC could quickly develop resistance to carbendazim. We identified two point mutations in the *Tub2* gene, including one novel point mutation from *F. proliferatum*, a temperature-susceptible FSC. Our results also provided a comprehensive analysis of mechanisms involved in FSC carbendazim resistance. By comparing transcriptome data for SJ51 and SJ51M with and without carbendazim treatment, genes related to carbendazim response and drug resistance were identified. These genes were involved in production of detoxification enzyme, drug efflux transporters as well as response to stress.

## Methods

### Sensitivity of FSC to carbendazim

Thirty-five single-spore isolates of FSC (Additional file 1: Table S1) were collected and recovered from 2012 to 2013 in the southern part of China that encompass major sugarcane production, including Fujian, Guangxi, Guangdong and Yunnan [8]. Carbendazim (97% a.i.; Yuanye; Shanghai, China) was dissolved in 0.1 M hydrochloric acid and adjusted to 10 mg a.i. mL^− 1^ as a stock solution to produce PDA medium (Hopebio; Qingdao, China) amended with 0, 0.5, 0.6, 0.7, 0.8, 0.9, 1.0, 1.1 or 1.2 μg a.i. mL^− 1^ carbendazim according to our preliminary results. A 5 mm diameter mycelial plug taken from the leading edge of a 3-day-old colony of each isolate was placed in the center of a 90 mm plate containing PDA medium amended with different carbendazim concentrations. Plates were incubated at 28 °C for 3 days in the dark, and the radial growth (colony diameter) of each isolate was measured in two perpendicular directions, with the original mycelial plug diameter (5 mm) subtracted from the measurement. Three replicate plates were used for each concentration and the experiment was repeated three times. For each isolate, the average radial colony growth was used to calculate the percent inhibition of mycelial growth and then a linear equation describing the percent inhibition of mycelial growth and the log_10_ of the fungicide concentration of each isolate was obtained. [12, 52] The EC_50_ was calculated according to the linear equation.

### Development of FSC mutants resistant to carbendazim in vitro

After measuring radial growth of colonies, the plates were used to obtain resistant mutants induced by carbendazim. Carbendazim-resistant mutants showed rapid growth in a fan-shaped region on the edge of the colony. The resistant phenotype strains in the fan-shaped region were transferred to fungicide-free PDA medium and continuously sub-cultured for 10 generations [18, 28]. The sensitivity of resistant strains was measured as described above.

### Characteristics of carbendazim mutants

To determine temperature sensitivity, mutant strains and wild type counterparts were used to assess the ability to grow at various temperatures on PDA medium with or without carbendazim. A 5 mm diameter mycelial plug taken from the leading edge of a 3-day-old colony of each isolate was placed on a plate containing PDA medium amended with carbendazim at 0, 1.2 or 1.9 μg a.i. mL^− 1^. Plates were incubated at 15 °C, 28 °C, 34 °C and 37 °C for 5 days in the dark, and the mycelial growth was recorded for each plate. Three replicate plates were used for each concentration and the experiment was performed three times. The radial growth (colony diameter) of each isolate cultured at 28 °C for 5 days without carbendazim treatment was measured in two perpendicular directions (with the original mycelial plug diameter subtracted from measurement) to analyze the fitness of mutants and the corresponding wild type strain. For pathogenicity assays, the conidia concentration of the wild type and mutants was adjusted to 1 × 10^6^ conidia per mL. Each strain was micro-injected into 15 healthy sugarcane plants and injection of water was used as a control. The symptoms were observed at 10 days after injection.

### DNA extraction, cloning and sequence analysis of the *Tub2* gene

Three-day old mycelium from resistant mutants and wild type strains were cultured in PDB medium (Hopebio; Qingdao, China) and collected for extraction of DNA using the CTAB method [53]. The specific primers FVER_05465F (5’-AGCGGCCAGTTAT TTCAGCA-3′), FVER_05465R (5’-GCCGATTTCTCTCCTCCTTCTC-3′), FVER_0 9254F (5’-TCCAATCCCTCTAGCCCTCG-3′) and FVER_09254R (5’-TCCTCGACA ACTTCACCACG-3′) were designed to amplify complete coding sequence (CDS) of *Tub2* gene based on the genome sequence of *F. verticillioides* CNO-1. FPRO_14041 and FPRO_07779 are derived from the genome of *F. proliferatum* YN41 and the amplified primers are identical to the FVER_0 9254 and FVER_05465, respectively. Three biological replicates of each strain used for DNA extraction and the PCR reactions were conducted three times independently for each sample. The amplified PCR products were purified using a PCR Purification Kit (TIANGEN; Beijing, China), ligated into the pMD18-T Vector (TaKaRa Biotech; Dalian, China), and then sequenced by Sangon (Guangzhou, China). The exon sequences of the *Tub2* gene were translated into amino acid sequences and aligned using DNAMAN8.0 software (Lynnon Biosoft; USA).

### Total RNA extraction, construction of cDNA library and Illumina sequencing

To explore the molecular basis of carbendazim resistance, the highly resistant mutant SJ51M and its wild type counterpart SJ51 were used. A 5 mm diameter mycelial plug taken from the leading edge of each colony was aseptically transferred to 100 mL of PDB and cultured at 220 rpm for 48 h at 28 °C in the dark. Then, carbendazim at the EC_50_ concentration (1.87 a.i. mL^− 1^ for SJ51M; 0.61 a.i. mL^− 1^ for SJ51) was added to the PDB medium. After 6 h incubation, the mycelia were collected, frozen in liquid nitrogen and stored at − 80 °C. Untreated samples were used as a control.

Total RNA was extracted and purified from three biological replicates of each treatment resulting in 12 samples using Quick-RNATM Miniprep according to the manufacturer’s instructions (Zymo Research, USA). The integrity and quality of the purified RNA were assessed by measuring the absorbance at 260/280 nm (A260/A280) and 1% agarose gel electrophoresis. To improve reliability and decrease the likelihood of biological error, equal amounts of total RNA from three biological replicates were pooled for Illumina deep RNA sequencing [23]. Sequencing libraries were constructed using NEBNext® Ultra™ RNA Library Prep Kit for Illumina® (NEB, USA) according to the manufacturer’s recommendations and index codes were added to attribute sequences to each sample. PCR products were purified (AMPure XP system) and the library quality was assessed on an Agilent Bioanalyzer 2100 system. Finally, the library preparations were sequenced on the Illumina Hiseq 2500 platform and paired-end reads were generated. Both library construction and sequencing were performed at BioMarker (Beijing, China).

### Reads mapping to the reference genome

Raw data (raw reads) of fastq format were processed through in-house perl scripts. In this step, reads containing adapter, poly-N and low-quality reads were removed from the raw data to obtain clean reads that were then mapped to our sequenced genome *F. verticillioides* CNO-1 using Tophat software (V2.0) by default parameters [54]. Only reads with a perfect match or one mismatch were further analyzed and annotated based on the reference genome.

### Gene expression analysis

DESeq provides statistical routines for determining differential expression in digital gene expression data using a model based on the negative binomial distribution. Fragments Per Kilobase of exon model per Million fragments mapped (FPKM) were used to estimate gene expression levels. DEGs analysis of two groups was performed using the DESeq R package (1.10.1) [55]. The resulting *P* values were adjusted using Benjamini and Hochberg’s approach for controlling the false discovery rate (FDR). Genes with FDR ≤ 0.05 and an absolute value of log_2_ (fold change) ≥2 were set as the threshold for significantly differential expression. GO enrichment analysis of the DEGs was implemented using GOseq R packages based on Wallenius non-central hyper-geometric distribution, which can adjust for gene length bias in DEGs [56]. Kyoto Encyclopedia of Genes and Genomes (KEGG) pathway enrichment analysis of the DEGs was performed using KOBAS software [57].

### qRT-PCR analysis

Total RNA (1 μg) from each sample was reverse transcribed using a PrimeScript™ RT reagent Kit with gDNA Eraser according to the manufacturer’s instructions (TaKaRa; Dalian, China). Primers were designed using Oligo software v.7.37 and the specificity was confirmed by BLAST analysis against the *F. verticillioides* CNO-1 genome. The sequences of the primers are listed in Additional file 8: Table S6. All qRT-PCR reactions were conducted in a LightCycler 480 thermocycler (Roche) with a 20 μl reaction volume using SYBR® Premix Ex Taq™ II (TaKaRa; Dalian, China), as per the manufacturer’s instructions. Melting curves were generated at the end of each PCR cycle to confirm the absence of nonspecific products in the reaction. Three biological replicate samples from each treatment were used for qRT-PCR analysis, and the reactions were performed in triplicate. To exclude the presence of contamination, a negative control containing no template (add sterile water) was included in all reactions. A 2^-ΔΔCt^ algorithm was used to evaluate the relative fold change in the expression of the each gene using the *act1* (actin) gene as an endogenous control. [58, 59] The data were analyzed using LightCycler® 480 software version 1.5.1 (Roche). Pearson’s correlation coefficients were calculated to evaluate the correlation of gene expression obtained by RNA-seq and qRT-PCR using Origin 9.0 software (Origin Lab).

## Additional files

Additional file 1: **Table S1.** Carbendazim sensitivity of FSC isolates from sugarcane. The carbendazim EC_50_ values for the 35 isolates ranged from 0.5097 to 0.6941 μg a.i. mL^− 1^ with an average EC_50_ of 0.5957 μg a.i. mL^− 1^. (DOCX 19 kb)

Additional file 2: **Table S2.** Carbendazim sensitivity of the resistant mutants. Five mutants had higher resistance to carbendazim with EC_50_ over 1.0 μg a.i. mL^− 1^, whereas another 13 mutants had EC_50_ values that were similar to those for the wild type. (DOCX 17 kb)

Additional file 3: **Figure S1.** Alignments of the the *Tub2* amino acid sequences from resistant mutants and their wild-type strains. The consistent sequences are indicated by a blue background. The amino acid substitutions at positions: T351I between HC30 and HC30M over FPRO_07779, and Q134L between SJ51 and SJ51M over FVER_09254 were indicated by a white background. (JPG 945 kb)

Additional file 4: **Table S3.** Effects of temperature on mycelial growth of resistant mutants and their wild-types amended with carbendazim. After culturing for 5 days, SJ51M grew at all tested temperatures on PDA with carbendazim, but wild type SJ51 failed to grow. Neither the mutant HC30M nor its wild type counterpart HC30 grew at 15 °C on PDA medium with carbendazim but did grow on PDA without carbendazim. (DOCX 17 kb)

Additional file 5: **Figure S2.** Characteristics of carbendazim mutants and their wild types. Radial growth (A) and colony morphology (B) of carbendazim mutants and their wild types grown at 28 °C for 5 days. The radial growth (colony diameter) and colony morphology did not show significant difference of mutants SJ51M and HC30M compared with their wild types SJ51 and HC30. Error bars represent SD (*n* = 9). (C) Pathogenicity test of the wild-types and their mutants. Each strain was inoculated by micro-injection with 1 × 10^6^ conidia mL^− 1^. The typical symptoms of growing point rot were observed after 10 days inoculation, while the control remained asymptomatic. Sterile water was used as a control. (TIF 2710 kb)

Additional file 6: **Table S4.** Percentages of reads mapped to the reference genome. TopHat2 tools soft were used to map with reference genome *F. verticillioides* CNO-1 by default parameters and over 75% of the total reads mapped to the genome. (DOCX 16 kb)

Additional file 7: **Table S5.** GO functional enrichment of the DEGs in SJ51M and its corresponding wild type strain SJ51. SJ51_C and SJ51M_C represented without carbendazim treatment, while SJ51_E and SJ51M_E represented exposed to carbendazim treatment. (XLSX 47 kb)

Additional file 8: **Table S6.** List of primers used for the qRT-PCR analysis. Ten genes related to transmembrane transport, oxidoreductase activity, response to stress and the target gene of carbendazim were validated via qRT-PCR analysis. (DOCX 16 kb)

## Abbreviations

a.i.: active ingredient
CDS: coding sequence
DEGs: differentially expressed genes
EC_50_: the fungicide concentration that inhibits 50% of mycelial growth
FDR: false discovery rate
FPKM: fragments per kilobase of exon model per million fragments mapped
FSC: *Fusarium* species complex
GO: gene ontology
GST: glutathione S-transferase
Hsp: heat shock protein
KEGG: Kyoto Encyclopedia of Genes and Genomes
MBC: methyl benzimidazole carbamate
MFS: major facilitator superfamily
P450s: cytochrome P450 monooxygenases
PDA: potato dextrose agar
PDB: potato dextrose broth
qRT-PCR: quantitative real-time PCR
RNA-seq: RNA sequencing
ROS: reactive oxygen species
SRA: Sequence Read Archive
*Tub2*: β-tubulin 2
