# Supplementary material for: Molecular characterization of carbendazim resistance of Fusarium species complex that causes sugarcane pokkah boeng disease
Source: BMC Genomics. 2019 Feb 7;20:115. doi: 10.1186/s12864-019-5479-6 (PMC6367828; doi:10.1186/s12864-019-5479-6)
Supplement: Supplementary file 1 — Table S1. Carbendazim sensitivity of FSC isolates from sugarcane. The carbendazim EC50 values for the 35 isolates ranged from 0.5097 to 0.6941 μg a.i. mL− 1 with an average EC50 of 0.5957 μg a.i. mL− 1. (DOCX 19 kb) [file 12864_2019_5479_MOESM1_ESM.docx]

**Additional file 1: Table S1.** Carbendazim sensitivity of FSC isolates from sugarcane.

| Isolate | Location | EC_50_  (μg a.i. mL^-1^) | Species^a^ | Genbank  no. |
| --- | --- | --- | --- | --- |
| CNO-1 | Chongzuo, Guangxi | 0.6139 | *F. verticillioides* | KJ629564 |
| HC13 | Hechi, Guangxi | 0.6389 | *F. verticillioides* | KJ629521 |
| HC35 | Hechi, Guangxi | 0.5369 | *F. verticillioides* | KJ629530 |
| HC34 | Hechi, Guangxi | 0.5888 | *F. verticillioides* | KJ629529 |
| HC01 | Hechi, Guangxi | 0.6298 | *F. verticillioides* | KJ629517 |
| HC30 | Hechi, Guangxi | 0.5893 | *F. proliferatum* | KJ629527 |
| HC11 | Hechi, Guangxi | 0.5340 | *F. verticillioides* | KJ629519 |
| GX28 | Laibing, Guangxi | 0.5922 | *F. verticillioides* | KJ629531 |
| GX09 | Liucheng, Guangxi | 0.6321 | *F. verticillioides* | KJ629535 |
| LC10 | Liucheng, Guangxi | 0.5175 | *F. verticillioides* | KJ629536 |
| LC05 | Liucheng, Guangxi | 0.5400 | *F. verticillioides* | KJ629533 |
| LC15 | Liucheng, Guangxi | 0.6465 | *F. verticillioides* | KJ629537 |
| BS37 | Baoshan, Yunnan | 0.6399 | *F. verticillioides* | KJ629509 |
| BS33 | Baoshan, Yunnan | 0.6142 | *F. verticillioides* | KJ629506 |
| BS40 | Baoshan, Yunnan | 0.5436 | *F. verticillioides* | KJ629512 |
| DH30 | Baoshan, Yunnan | 0.6339 | *F. verticillioides* | KJ629485 |
| DH19 | Dehong, Yunnan | 0.6020 | *F. verticillioides* | KJ629479 |
| DH24 | Dehong, Yunnan | 0.5959 | *F. verticillioides* | KJ629480 |
| DH05 | Dehong, Yunnan | 0.6254 | *F. verticillioides* | KJ629470 |
| DH12 | Dehong, Yunnan | 0.5613 | *F. verticillioides* | KJ629475 |
| YN54 | Gengma, Yunnan | 0.5839 | *F. verticillioides* | KJ629487 |
| GM58 | Gengma, Yunnan | 0.5502 | *F. verticillioides* | KJ629491 |
| SJ45 | Shuangjiang, Yunnan | 0.5894 | *F. verticillioides* | KJ629494 |
| SJ51 | Shuangjiang, Yunnan | 0.6111 | *F. verticillioides* | KJ629500 |
| SJ47 | Shuangjiang, Yunnan | 0.5464 | *F. verticillioides* | KJ629496 |
| SJ67 | Shuangjiang, Yunnan | 0.5556 | *F. verticillioides* | KJ629504 |
| CT46 | Changtai, Fujian | 0.5097 | *F. verticillioides* | KJ629516 |
| FZ15 | Fuzhou, Fujian | 0.6165 | *F. verticillioides* | KJ629561 |
| FZ10 | Fuzhou, Fujian | 0.6177 | *F. verticillioides* | KJ629556 |
| FZ04 | Fuzhou, Fujian | 0.6245 | *F. verticillioides* | KJ629551 |
| FZ12 | Fuzhou, Fujian | 0.6941 | *F. verticillioides* | KJ629558 |
| LW54 | Zhanzhou, Fujian | 0.5919 | *F. verticillioides* | KJ629542 |
| LW67 | Zhangzhou, Fujian | 0.6316 | *F. verticillioides* | KJ629546 |
| FN22 | Zhanjiang, Guangdong | 0.6343 | *F. verticillioides* | KJ629567 |
| FN29 | Zhanjiang, Guangdong | 0.6150 | *F. verticillioides* | KJ629570 |
| Mean | - | 0.5957 | - | - |

^a^ Based on the results of whole genome sequence alignment, HC30 was corrected to *F. proliferatum***.**

The carbendazim EC_50_ values for the 35 isolates ranged from 0.5097 to 0.6941 μg a.i. mL^–1^ with an average EC_50_ of 0.5957 μg a.i. mL^–1^.
