# Supplementary material for: Molecular characterization of carbendazim resistance of Fusarium species complex that causes sugarcane pokkah boeng disease
Source: BMC Genomics. 2019 Feb 7;20:115. doi: 10.1186/s12864-019-5479-6 (PMC6367828; doi:10.1186/s12864-019-5479-6)
Supplement: Supplementary file 2 — Table S2. Carbendazim sensitivity of the resistant mutants. Five mutants had higher resistance to carbendazim with EC50 over 1.0 μg a.i. mL− 1, whereas another 13 mutants had EC50 values that were similar to those for the wild type. (DOCX 17 kb) [file 12864_2019_5479_MOESM2_ESM.docx]

**Additional file 2: Table S2.** Carbendazim sensitivity of the resistant mutants.

| Resistant mutants^a^ | Concentration  (μg a.i. mL^-1^ )^b^ | EC_50_  (μg a.i. mL^-1^)^c^ |
| --- | --- | --- |
| SJ51M | 0.8 | 1.8689 |
| FZ04M | 0.7 | 0.9139 |
| DH19M1 | 0.6 | 0.8695 |
| DH19M2 | 0.6 | 0.8515 |
| DH19M3 | 0.7 | 0.5549 |
| HC30M | 0.9 | 1.0958 |
| FZ15M | 0.7 | 1.1235 |
| HC35M1 | 0.6 | 0.6246 |
| HC35M2 | 0.7 | 0.7697 |
| YN54M | 0.7 | 1.0059 |
| HC34M | 0.7 | 0.5427 |
| FZ10M | 0.7 | 0.6030 |
| LW54M1 | 0.5 | 0.6527 |
| LW54M2 | 0.6 | 0.6992 |
| CT46M | 0.6 | 0.7407 |
| GX28M | 0.7 | 0.6744 |
| FN29M | 0.8 | 0.6719 |
| FN22M | 0.8 | 1.0452 |

^a^ The strains in the fan-shaped region on the edge of the colony induced by carbendazim.

^b^ Carbendazim concentrations to produce resistant mutants.

^c^ EC_50_ values of the resistant mutants to carbendazim after continuous sub-cultured for 10 generations on fungicide-free PDA medium.

Five mutants had higher resistance to carbendazim with EC_50_ over 1.0 μg a.i. mL^-1^, whereas another 13 mutants had EC_50_ values that were similar to those for the wild type.
