# Supplementary figures and images for: Molecular characterization of carbendazim resistance of Fusarium species complex that causes sugarcane pokkah boeng disease
Source: BMC Genomics. 2019 Feb 7;20:115. doi: 10.1186/s12864-019-5479-6 (PMC6367828; doi:10.1186/s12864-019-5479-6)

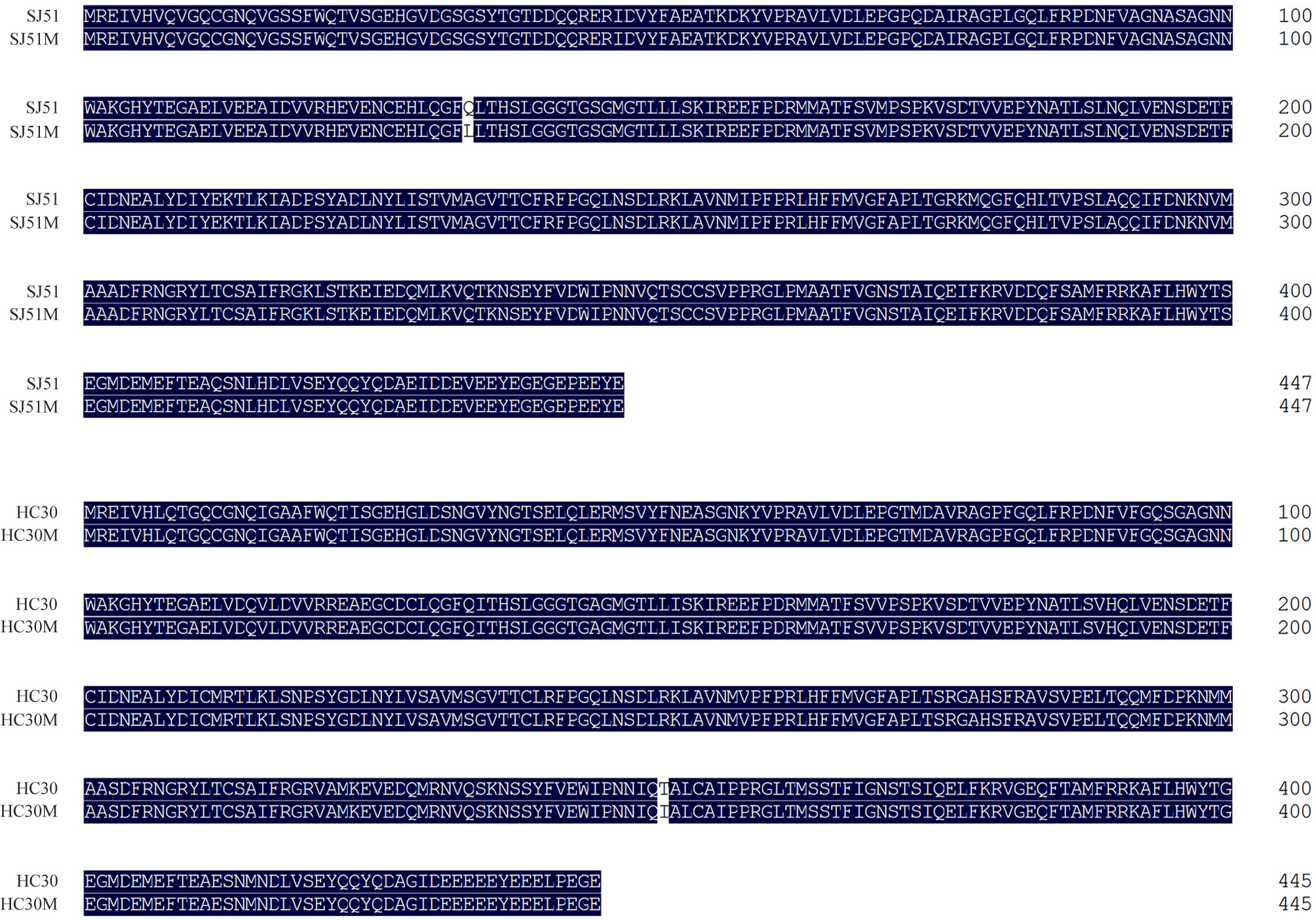

Supplement: Supplementary file 3 — Figure S1. Alignments of the the Tub2 amino acid sequences from resistant mutants and their wild-type strains. The consistent sequences are indicated by a blue background. The amino acid substitutions at positions: T351I between HC30 and HC30M over FPRO_07779, and Q134L between SJ51 and SJ51M over FVER_09254 were indicated by a white background. (JPG 945 kb) [file 12864_2019_5479_MOESM3_ESM.jpg]

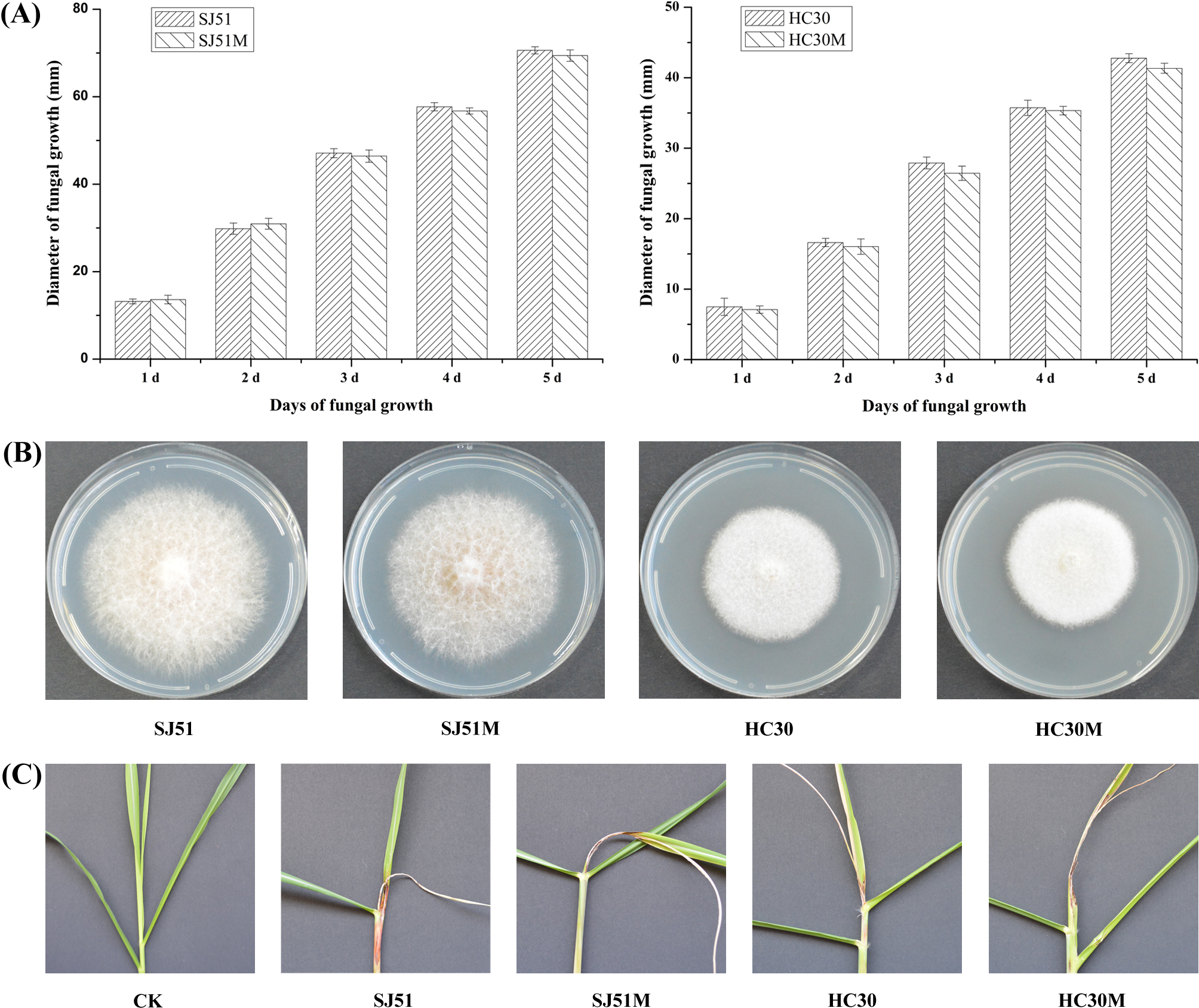

Supplement: Supplementary file 5 — Figure S2. Characteristics of carbendazim mutants and their wild types. Radial growth (A) and colony morphology (B) of carbendazim mutants and their wild types grown at 28 °C for 5 days. The radial growth (colony diameter) and colony morphology did not show significant difference of mutants SJ51M and HC30M compared with their wild types SJ51 and HC30. Error bars represent SD (n = 9). (C) Pathogenicity test of the wild-types and their mutants. Each strain was inoculated by micro-injection with 1 × 10^6^ conidia mL− 1. The typical symptoms of growing point rot were observed after 10 days inoculation, while the control remained asymptomatic. Sterile water was used as a control. (TIF 2710 kb) [file 12864_2019_5479_MOESM5_ESM.tif]
