# Supplementary material for: Molecular characterization of carbendazim resistance of Fusarium species complex that causes sugarcane pokkah boeng disease
Source: BMC Genomics. 2019 Feb 7;20:115. doi: 10.1186/s12864-019-5479-6 (PMC6367828; doi:10.1186/s12864-019-5479-6)
Supplement: Supplementary file 4 — Table S3. Effects of temperature on mycelial growth of resistant mutants and their wild-types amended with carbendazim. After culturing for 5 days, SJ51M grew at all tested temperatures on PDA with carbendazim, but wild type SJ51 failed to grow. Neither the mutant HC30M nor its wild type counterpart HC30 grew at 15 °C on PDA medium with carbendazim but did grow on PDA without carbendazim. (DOCX 17 kb) [file 12864_2019_5479_MOESM4_ESM.docx]

**Additional file 4:** **Table S3.** Effects of temperature on mycelial growth of resistant mutants and their wild-types amended with carbendazim.

| Temp  (°C) | Carbendazim concn  (μg a.i. mL^-1^)^a^ | Mycelial growth of FSC isolations (mm) | | | |
| --- | --- | --- | --- | --- | --- |
|  |  | SJ51 | SJ51M | HC30 | HC30M |
| 15 | 0 | 38.68 | 38.45 | 36.16 | 35.78 |
|  | EC_50_ | 0 | 10.87 | 0 | 0 |
| 28 | 0 | 70.61 | 69.41 | 42.78 | 41.34 |
|  | EC_50_ | 0 | 36.66 | 0 | 23.37 |
| 34 | 0 | 63.09 | 62.10 | 32.08 | 31.89 |
|  | EC_50_ | 0 | 34.15 | 0 | 20.32 |
| 37 | 0 | 17.25 | 17.92 | 2.40 | 2.11 |
|  | EC_50_ | 0 | 12.03 | 0 | 2.34 |

^a^ The carbendazim concentration was adjusted based on the EC_50_ value. The carbendazim concentration used for SJ51 and SJ51M was 1.9 μg a.i. mL^-1^; the carbendazim concentration used for HC30 and HC30M was 1.2 μg a.i. mL^-1^.

SJ51M grew at all tested temperatures on PDA with carbendazim, but wild type SJ51 failed to grow. Neither the mutant HC30M nor its wild type counterpart HC30 grew at 15 °C on PDA medium with carbendazim but did grow on PDA without carbendazim.
