# Supplementary material for: Molecular characterization of carbendazim resistance of Fusarium species complex that causes sugarcane pokkah boeng disease
Source: BMC Genomics. 2019 Feb 7;20:115. doi: 10.1186/s12864-019-5479-6 (PMC6367828; doi:10.1186/s12864-019-5479-6)
Supplement: Supplementary file 6 — Table S4. Percentages of reads mapped to the reference genome. TopHat2 tools soft were used to map with reference genome F. verticillioides CNO-1 by default parameters and over 75% of the total reads mapped to the genome. (DOCX 16 kb) [file 12864_2019_5479_MOESM6_ESM.docx]

**Additional file 6:** **Table S4.** Percentages of reads mapped to the reference genome.

| Sample name | SJ51_C | SJ51_E | SJ51M_C | SJ51M_E |
| --- | --- | --- | --- | --- |
| Total Reads | 67,793,314 | 50,901,658 | 59,321,854 | 48,228,916 |
| Mapped Reads | 52,759,268 (77.82%) | 38,929,471 (76.48%) | 45,320,417 (76.40%) | 36,353,996 (75.38%) |
| Uniq Mapped | 50,859,108 (75.02%) | 37,699,060 (74.06%) | 44,320,514 (74.71%) | 35,597,099 (73.81%) |
| Multiple Map | 1,900,160 (2.80%) | 1,230,411 (2.42%) | 999,903 (1.69%) | 756,897 (1.57%) |
| Reads Map to '+' | 26,355,576 (38.88%) | 19,450,108 (38.21%) | 22,662,220 (38.20%) | 18,169,683 (37.67%) |
| Reads Map to '-' | 26,403,692 (38.95%) | 19,479,363 (38.27%) | 22,658,197 (38.20%) | 18,184,313 (37.70%) |

SJ51_C and SJ51M_C represented without carbendazim treatment, while SJ51_E and SJ51M_E represented exposed to carbendazim treatment.

TopHat2 tools soft were used to map with reference genome *F. verticillioides* CNO-1 by default parameters and over 75 % of the total reads mapped to the genome.
