# Supplementary material for: Molecular characterization of carbendazim resistance of Fusarium species complex that causes sugarcane pokkah boeng disease
Source: BMC Genomics. 2019 Feb 7;20:115. doi: 10.1186/s12864-019-5479-6 (PMC6367828; doi:10.1186/s12864-019-5479-6)
Supplement: Supplementary file 8 — Table S6. List of primers used for the qRT-PCR analysis. Ten genes related to transmembrane transport, oxidoreductase activity, response to stress and the target gene of carbendazim were validated via qRT-PCR analysis. (DOCX 16 kb) [file 12864_2019_5479_MOESM8_ESM.docx]

**Additional file 8: Table S6. List of primers used for the qRT-PCR analysis.**

| Gene ID | Forward (5'-3') | Reverse (5'-3') | Function description |
| --- | --- | --- | --- |
| FVER_02883 | TCGATCTTGATGCCAACGGTA | GCTCGATCTCCTCCTTGGACA | Heat shock 70 kDa protein |
| FVER_03030 | CGTGAGGAGAAGCTGAAGTATG | GAGACCAGTTCGACACCAATAG | ATP-binding multidrug cassette transport protein |
| FVER_03117 | TTGAAGTCGGCGAGAAGAAG | GGCAGTAGGAACCTCGTTAAG | Heat shock protein 60 |
| FVER_09151 | CAGAGAAGTCCAAGACCGTTAG | GAAAGTGAAGTAGCGGGAGAA | Related to heat shock protein 30 |
| FVER_09254 | CCAGCTTGTCGAGAACTCCGAT | ACGCCAGCCATAACTGTCGAG | Beta-tubulin 2 |
| FVER_09560 | TGGCCAATATCTGGCTTGAC | CGAGCATCTCTCCGTTCTTTAC | ABC transporter CDR4 |
| FVER_10345 | AGGTTCTCTTCCTCGTTGACCC | TCTCCTCAAGCTCGAAGTCC | Heat shock protein 90 |
| FVER_11009 | AGTACCTCACCGGCATCGTC | TTAGCGTGGAGACTGAAATTCCC | MFS-type transporter |
| FVER_11010 | ATCACAGCCATGCCGACCT | ACCCTCTTCTGCGCTAGTCCC | Thioredoxin |
| FVER_11289 | GCTCGTCATGTCGTGTATCTATC | CGGCATAGTGAAGCCAGTATAA | Multidrug resistance protein CDR2 |
| Reference gene | GAGAACGAGCGTGTCTTGATTGAGCC | TTTCCTCCGCAGAATGAAGAAGGACTC | Actin gene |

Ten genes related to transmembrane transport, oxidoreductase activity, response to stress and the target gene of carbendazim were validated via qRT-PCR analysis.
